# Edematous changes and nail ulcers in hand transplant after SARS‐COV‐2 infection: Unraveling infection or rejection?

**DOI:** 10.1002/kjm2.12941

**Published:** 2025-01-15

**Authors:** Lee‐Moay Lim, Yue‐Chiu Su, Yur‐Ren Kuo

**Affiliations:** ^1^ Division of Nephrology, Department of Internal Medicine Kaohsiung Medical University Hospital, Kaohsiung Medical University Kaohsiung Taiwan; ^2^ School of Medicine, College of Medicine Kaohsiung Medical University Kaohsiung Taiwan; ^3^ Department of Pathology Kaohsiung Medical University Hospital, Kaohsiung Medical University Kaohsiung Taiwan; ^4^ Department of Pathology Kaohsiung Medical University Kaohsiung Taiwan; ^5^ Department of Plastic & Reconstructive Surgery Kaohsiung Medical University Hospital, Kaohsiung Medical University Kaohsiung Taiwan

A vascularized composite allotransplantation (VCA) of the upper extremity is a proven treatment option for selected patients who have acquired upper limb loss. After transplantation, transplant recipients are at high risk of contracting infections because of immunosuppressive therapies. As a result, maintaining the health status of VCA patients is challenging, since organ rejection is triggered by T‐cell recognition of antigens, either because of infection or immune activation.^1^ In VCA allografts, acute rejection is usually detected directly from the graft, which partly explains the high diagnosis rate. As there are no assays for graft rejection for monitoring purposes, infection of the allograft could make diagnosis more difficult.^2^ The present report describes a hand transplant recipient who developed erythematous plaques and nail ulcers after the SARS‐CoV‐2 infection, and eventually whose transplanted hand had to be removed.

These 53‐year‐old males who suffered from crushed injury of his left upper limb received below‐elbow allograft hand transplantation in 2014. Following his allotransplantation, he took oral immunosuppressive agents including mychophenolate mofetil, tacrolimus, and prednisolone, as well as topical immunosuppressive ointment. He presented with edematous change over his allotransplanted hand in 2022 (Figure 1A). Upon inspection, erythematous plaques were found over his proximal nail fold of left fingers, with necrotic change and pus like discharge over his third and fourth finger (Figure 1B). Prior to the appearance of the plaques, he was infected with SARS‐CoV‐2, so his immunosuppressive agents were reduced to aid his recovery. Based on the pathological findings of the fourth finger skin biopsy, the plaques were identified as sub‐corneal pustules free of fungal elements (Figure 1A,B). Chronic rejection exhibiting intimal proliferation in vessels was noted in the subcutaneous fat in the forearm skin biopsy (Figure 1C–F). During the follow‐up, pale skin, graft edema, and rash were found (Figure 1C), with acute and chronic rejection proven from biopsy. The titration of immunosuppressive agents did not yield satisfactory results to reverse the rejection. Due to the patient's immunocompromised status and the uncontrolled rejection, the transplanted hand was amputated.

**FIGURE 1:**
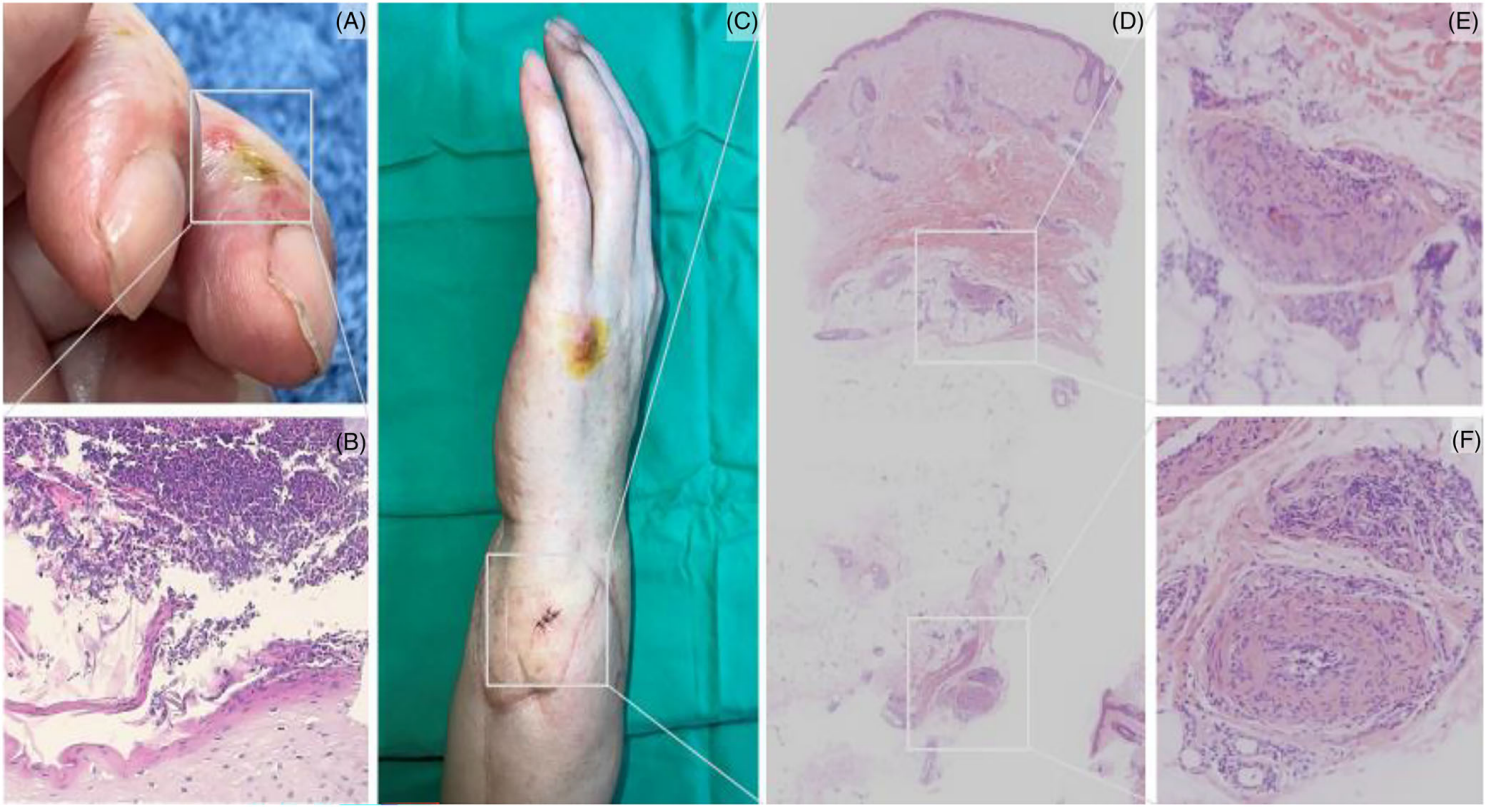
The fourth finger revealed a sub‐corneal pustule without fungal elements (A and B). The forearm skin showed solar elastosis, decreased number and atrophy of adnexal structures, dermal fibrosis and intimal proliferation in vessels at the subcutaneous fat. Chronic rejection 1 is considered (C–F). Pale skin, graft edema, and rash were found (C).

The use of immunosuppressants increases the risk of contracting SARS‐CoV‐2 in transplant recipients. SARS‐CoV‐2 recovery may be improved with the reduction of immunosuppressants, but damage to transplanted hand resulting from inflammatory responses is significantly more detrimental. Skin rejection typically manifests as a maculopapular erythematous rash on the upper forearm and dorsum of the hands, sparing the palm and nails.^3^ Atypical rejection characterized by nail changes and palmar involvement were rare, as described by Schneeberger et al. in their study.^3^ An immune dysregulation induced by SARS‐CoV‐2 can lead to fatal immune responses.^4^ The high rejection rate among renal and pancreatic transplant recipients with COVID‐19 suggests widespread immune reactivation despite immunosuppressive therapy.^5^

Our patient's immunocompromised status makes him particularly susceptible to coinfections. A prompt graft skin biopsy may be able to provide the most accurate early diagnosis. Consequently, the allotransplanted hand was removed because of uncontrolled rejection and infection, and the patients recovered uneventfully. In our experience, closely monitoring VCA patients with SARS‐CoV‐2 infection can result in an earlier rejection detection, which may lead to the patient surviving their allotransplant.

## CONFLICT OF INTEREST STATEMENT

The authors declare no conflict of interest.

## Data Availability

The data that support the findings of this study are available on request from the corresponding author. The data are not publicly available due to privacy or ethical restrictions.
